# Dinoflagellate–Bacteria Interactions: Physiology, Ecology, and Evolution

**DOI:** 10.3390/biology13080579

**Published:** 2024-07-31

**Authors:** Xiaohong Yang, Zijian Liu, Yanwen Zhang, Xinguo Shi, Zhen Wu

**Affiliations:** 1 Guangzhou Marine Geological Survey, Guangzhou 511458, China; 2 Shenzhen Branch, Guangdong Laboratory of Lingnan Modern Agriculture, Genome Analysis Laboratory of the Ministry of Agriculture and Rural Affairs, Agricultural Genomics Institute at Shenzhen, Chinese Academy of Agricultural Sciences, Shenzhen 518000, China; 3 Microbial Processes and Interactions (MiPI), TERRA Teaching and Research Centre, Gembloux Agro-Bio Tech, University of Liège, 5030 Gembloux, Belgium; 4 Department of Ocean Science and Engineering, Southern University of Science and Technology (SUSTech), Shenzhen 518055, China; 5 College of Biological Science and Engineering, Fuzhou University, Fuzhou 350108, China

**Keywords:** dinoflagellates, heterotrophic bacteria, co-evolution, nutrient exchange, pathogenic substances, chemical production, horizontal gene transfer

## Abstract

**Simple Summary:**

This review highlights the intricate interactions between two major constituents of marine ecosystems: dinoflagellates and heterotrophic bacteria. Dinoflagellates typically associate with a diverse array of heterotrophic bacteria, which are not limited to but predominantly consist of phyla such as Alphaproteobacteria, Gammaproteobacteria, and the *Cytophaga–Flavobacterium–Bacteroides* group. These bacterial communities engage with dinoflagellates in a multifaceted manner, including nutrient exchange, the secretion of pathogenic substances, and involvement in the synthesis of chemical entities. Crucially, the process of horizontal gene transfer from bacteria is a significant force sculpting the genomic architecture of dinoflagellates. The objective of this review is to shed light on the dynamic interactions between dinoflagellates and their bacterial partners, examining the relationship through a physiological, ecological, and evolutionary perspective.

**Abstract:**

Dinoflagellates and heterotrophic bacteria are two major micro-organism groups within marine ecosystems. Their coexistence has led to a co-evolutionary relationship characterized by intricate interactions that not only alter their individual behaviors but also exert a significant influence on the broader biogeochemical cycles. Our review commenced with an analysis of bacterial populations, both free-living and adherent to dinoflagellate surfaces. Members of Alphaproteobacteria, Gammaproteobacteria, and the Cytophaga–Flavobacterium–Bacteroides group are repeatedly found to be associated with dinoflagellates, with representation by relatively few genera, such as *Methylophaga*, *Marinobacter*, and *Alteromonas*. These bacterial taxa engage with dinoflagellates in a limited capacity, involving nutrient exchange, the secretion of pathogenic substances, or participation in chemical production. Furthermore, the genomic evolution of dinoflagellates has been profoundly impacted by the horizontal gene transfer from bacteria. The integration of bacterial genes into dinoflagellates has been instrumental in defining their biological characteristics and nutritional strategies. This review aims to elucidate the nuanced interactions between dinoflagellates and their associated bacteria, offering a detailed perspective on their complex relationship.

## 1. Introduction

Bacteria, archaea, and protists collectively form the majority of biomass in the vast expanse of our oceans [1,2]. These microscopic organisms, though often unseen, wield immense influence over marine ecosystems. They play different roles in their respective ecological niches. Protists mainly include primary producers (microalgae) and primary consumers. Bacteria are major decomposers. Archaea act as decomposers by degrading organic matter or engage in chemoautotrophy, contributing to primary production in environments devoid of sunlight [3]. Rather than existing in isolation, these tiny life forms actively interact with each other [4]. Marine ecosystems connect them at a range of spatial scales through interactions including mutualism, competition, antagonism, and so on. These interactions play a crucial role in driving the oceanic biogeochemical cycles, which, in turn, shape their biomass and overall dynamics [4,5,6].

In this review, we focus on interactions between two major groups of marine microbes: dinoflagellates and heterotrophic bacteria. Dinoflagellates dominate the ocean community as the second most abundant eukaryotic microalgae [7,8]. They provide sustenance for heterotrophic bacteria, which, in turn, play essential roles as primary consumers and decomposers within marine ecosystems [9,10]. By utilizing organic carbon produced by primary producers, the heterotrophic bacteria effectively remineralize a substantial portion of organic matter back into CO_2_ [11,12]. They also provide dinoflagellates with valuable nutrients and minerals [13]. Due to their ubiquity and diverse functionalities, marine heterotrophic bacteria significantly influence the biogeochemical cycles of essential elements. The coexistence of dinoflagellates and bacteria in common ocean habitats spans an impressive 400 million years [14]. Over evolutionary time scales, these two groups have fostered intricate interactions. Notably, many genes in dinoflagellate genomes appear to have been acquired from bacteria [15]. These genetic acquisitions likely contributed significantly to the diversity and success of dinoflagellates.

Understanding interactions between dinoflagellates and bacteria is of major significance in understanding oceanic nutrient fluxes and their co-evolutionary history. In this review, we provide a concise overview of known bacterial associations with dinoflagellates, revealing that these associations tend to be limited to a few specific genera. Building upon these associations, we investigate the microscopic environment shared by dinoflagellates and bacteria, illustrating how it influences their encounters. Additionally, we explore documented dinoflagellate–bacterium interactions, aiming to identify commonalities across these interactions. Finally, we engage in a discussion about how genes of bacterial origin shape the evolution of dinoflagellates.

## 2. Dinoflagellates Harbor Distinct Bacterial Communities

Dinoflagellates coexist with a multitude of heterotrophic bacteria, actively engaging in interactions within the marine environment, rather than existing in isolation [9]. This complex relationship contributes to the dynamic nature of the microbial ecosystem. These bacteria form associations with dinoflagellates that range from loose to tight within marine ecosystems. Notably, in cultures of dinoflagellates, an average of six bacteria attach to each dinoflagellate cell, although a significant number remain unattached [13]. Some bacteria even act as endosymbionts, residing inside the dinoflagellate cells [16,17]. This intimate association between dinoflagellates and bacteria poses challenges for isolating, maintaining, and studying axenic cultures of dinoflagellates.

**Figure 1:**
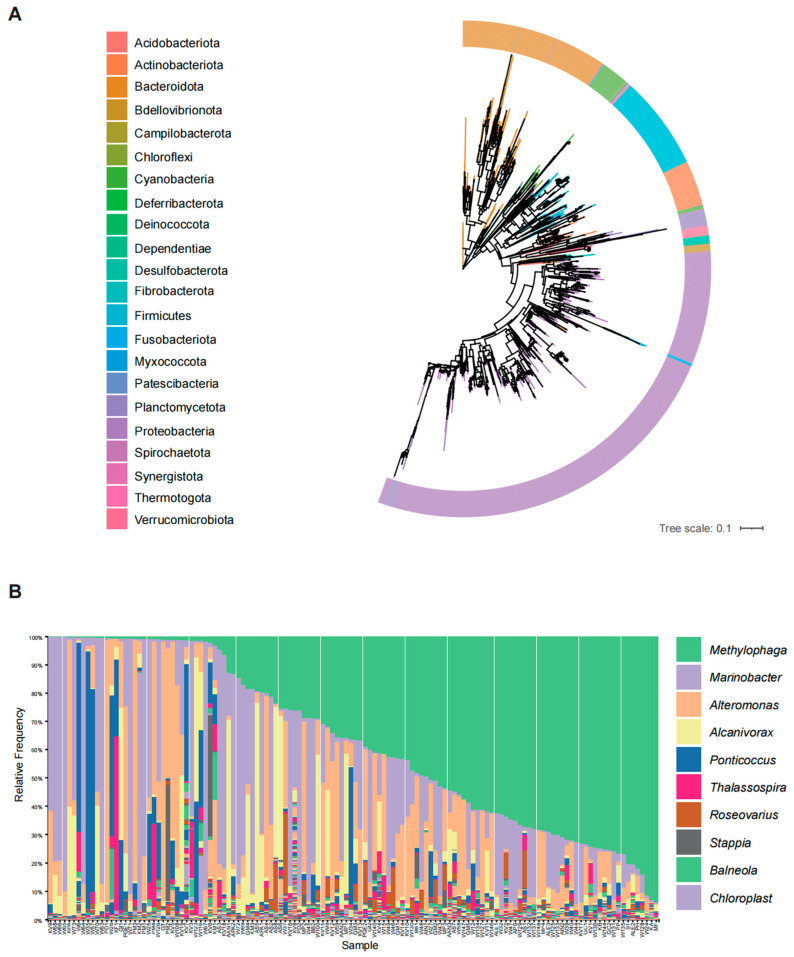
(**A**). The maximum-likelihood phylogenetic tree of 16S rRNA gene sequences of the bacterial phyla associated with 130 dinoflagellate cultures. Bacterial phyla are labeled in the corresponding-colored ring. (**B**). Relative abundance of the top 10 different bacterial genera associated with 130 dinoflagellate cultures. A total of 3359 features from 130 dinoflagellates (PRJNA771505, [7]) were aligned using MAFFT (Verision 7.471, [23]) and subsequently subjected to phylogenetic analysis via FastTree (Version 2.1.11, [24]). Silva database (Version 132, [25]) was employed for taxonomic annotation based on the Quantitative Insights Into Microbial Ecology version 2 (Qiime2) software [26] and these sequences were denominated at the phylum and genus levels.

The most common methods for studying bacteria associated with dinoflagellates include cultivation-based approaches and field-sample-based assessments. Cultivation-based methods provide critical insights into the metabolism of specific micro-organisms. For instance, research on the effects of epiphytic bacteria on the growth of the ciguatera-causing dinoflagellate, *Gambierdiscus* (*G*) *toxicus* R. Adachi & Y. Fukuyo, 1979, showed that the strain *Flavobacterium* sp. C1 significantly inhibited algal growth [18]. Conversely, the toxic dinoflagellate *Gymnodinium* (*Gy*) *catenatum* H.W. Graham, 1943 requires specific bacteria for survival and growth post-germination, from resting cyst germination to vegetative growth [19]. A molecular survey of bacterial assemblage was conducted from cultures of 144 harmful algal strains, including 130 dinoflagellates [7]. This study revealed the presence of 3357 bacterial taxa, including 22 phyla, 38 classes, 110 orders, 199 families, and 401 genera associated with these dinoflagellates (Figure 1). Proteobacteria, Bacteroidota, and, to a lesser extent, Firmicutes were predominant across all examined dinoflagellates (Figure 1A). Bacterial communities were dominated by a relatively small number of genera, most notably, the Gammaproteobacteria *Methylophaga*, *Marinobacter*, and *Alteromonas* (Figure 1B). Recent studies have identified the core microbiomes of various dinoflagellate cultures, including the Symbiodiniaceae dinoflagellates [20], *Gambierdiscus balechii* (*G*) S. Fraga, F. Rodríguez & I. Bravo, 2016 [21], *Karlodinium* (*K*) *veneficum* (D. Ballantine) J. Larsen, 2000 [7], and *Prorocentrum* (*P*) *cordatum* (Ostenfeld) J. D. Dodge, 1976 [22]. These findings indicate that Gammaproteobacteria and Alphaproteobacteria are the primary heterotrophic bacterial classes associated with dinoflagellates. The existence of a core microbiome suggests the significant ecological impact of bacteria on the functions and fitness of their host dinoflagellates.

Other research has focused on bacterial community dynamics during dinoflagellate blooms or in dinoflagellate-dominated phytoplankton communities. For instance, the composition and amount of free and attached bacterial communities was found to differ significantly during a dinoflagellate bloom, with the *Cytophaga–Flavobacterium* group, Gammaproteobacteria, and Alphaproteobacteria as the dominant taxa [27]. During a spring bloom of *Alexandrium* (*A*) *catenella* (Whedon & Kofoid) Balech, 1985, Gammaproteobacteria and Bacteroidetes were prevalent during the initial stage, while Alphaproteobacteria, Cyanobacteria, and Actinobacteria became more abundant during the onset and termination of the bloom [28]. Camarena-Gómez et al. (2020) observed a shift in microbial groups from a diatom-dominated bloom to a dinoflagellate-dominated bloom, noting that the increase in dinoflagellate abundance significantly influenced the structure and function of the associated bacterial communities. The dominant bacterial taxa shifted from copiotrophic bacteria (Flavobacteriia, Gammaproteobacteria, and Betaproteobacteria) during the diatom bloom to Alphaproteobacteria during the dinoflagellate bloom [29]. Even though 16S rRNA surveys of bacteria have uncovered a remarkable diversity of bacterial assemblages, there has been notable conservation across different algal strains, with the *Cytophaga–Flavobacterium–Bacteroides* (CFB) group, Gammaproteobacteria, and Alphaproteobacteria frequently reported in dinoflagellate blooms [28,30,31,32].

The overview of bacterial associations with dinoflagellates reveals that the heterotrophic bacterial species consistently observed with dinoflagellates are typically limited to a few phyla. The specific interactions between these bacteria and dinoflagellates, as well as their mutual influence, will be discussed in more detail in subsequent sections.

## 3. Dinoflagellates Recruit Bacteria through Dissolved Organic Matter (DOM)

In the marine environment, the prevailing paradigm is that autotrophs are responsible for carbon fixation, while heterotrophic bacteria play a crucial role in assimilating and decomposing a significant proportion of this carbon [9,10]. Dinoflagellates are a primary source of DOMs, which is essential for the growth and functioning of specific heterotrophic bacterial genera [33]. These bacteria interact with dinoflagellates through chemical exchange.

DOMs produced by dinoflagellates consists primarily of sugars, amino acids, nucleosides, and other small diffusible organic compounds, such as the organosulfur compound dimethylsulfoniopropionate (DMSP) and nucleosides [34,35,36]. The utilization of these compounds varies among bacteria associated with dinoflagellates. For instance, the *Silicibacter* sp. strain TM1040, isolated from the heterotrophic dinoflagellate *Pfiesteria piscicida* Steidinger & J. M. Burkholder, 1996 culture, showed a strong attraction to amino acids and DMSP metabolites, while it exhibited only a mild response to sugars and the tricarboxylic acid cycle intermediates [37]. The DOM derived from different dinoflagellate species varies, and they recruit differential representatives of bacterial families [29]. For instance, Lin et al. (2021) [36] observed significant differences in the richness and diversity of bacteria across different dinoflagellate cultures with varying DMSP contents. In another study by Han et al. (2021) [38], bacterial diversity, genome traits, and metabolic responses were assessed to determine the source and lability of DOM in a spring coastal bloom of *Akashiwo* (*Ak*) *sanguinea* (K. Hirasaka) Gert Hansen & Moestrup, 2000 using a metabolomic approach. The metabolomic data revealed that amino acids and dipeptides, such as isoleucine and proline, were preferentially taken up by *Polaribacter marinivivus* Park et al., 2015 and *Lentibacter algarum* Li et al., 2012, whereas nucleotides and nucleosides (such as adenosine and purine) were preferentially selected by *Litoricola marina* Choi et al., 2010 [38]. Overall, DOMs originating from dinoflagellates significantly influence the composition and functioning of the dinoflagellate-associated microbial community.

In a separate study, Osbeck et al. (2022) [39] conducted a transcriptional analysis of two heterotrophic bacterial isolates exposed to dinoflagellate DOMs, comparing their responses to control cultures. While both bacterial isolates exhibited similarities, such as the upregulation of genes related to Ton (resistance to bacteriophage T-one) and Tol (tolerance to colicins) transport systems that are involved in the uptake of nutrients and essential elements [40], they also displayed significant differences [39]. These variations included distinct gene regulation patterns and the presence of genes associated with membrane transport, motility, and photoheterotrophy [39]. These findings underscore the diverse strategies employed by different bacterial isolates when interacting with dinoflagellate-derived organic matters.

## 4. Growth-Promoting Metabolic Substances Generated by Bacteria for the Growth of Dinoflagellates

The interaction between bacteria and dinoflagellates often centers around bacterial production of vitamins essential for dinoflagellate species. The earliest documented correlation between vitamins and dinoflagellates dates to 1954, when *Gymnodinium splendens* Lebour, 1925 was found to require cobalamin (vitamin B12) for growth [41]. Vitamin B12 has been extensively studied in the context of dinoflagellate requirements. Recent field studies consistently reveal that many harmful-algal-bloom-forming dinoflagellate species have strict vitamin B requirements [42,43,44]. These requirements can be so high that they rapidly deplete vitamin B12 stocks in coastal waters, sometimes within days to hours [42]. For instance, blooms of dinoflagellates such as *Cochlodinium* (*C*) *polykrikoides* Margalef, 1961, *K. veneficum*, and *Prorocentrum* (*P*) *minimum* (Pavillard) J. Schiller, 1933 can reduce vitamin B1 and B12 concentrations by up to 90% to critically low levels [42].

In 2005, Croft and colleagues conducted a comprehensive literature survey on vitamin B12 requirements across 326 algal species, confirming its necessity in specific cases. Among the surveyed dinoflagellates, 26 out of 30 were unable to grow in a B12-deficient medium, highlighting the critical role of vitamin B12 for these organisms [45]. Additionally, researchers isolated a bacterial species from the genus *Halomonas* in the culture medium, which demonstrated the ability to synthesize vitamin B12 de novo [45]. When introduced to axenic cultures of dinoflagellates *Amphidinium* (*Am*) *operculatum* Claparède & Lachmann, 1859 and *Porphyridium purpureum* (Bory) K.M.Drew & R.Ross, 1965, this bacterium supported algal growth to the same extent as vitamin B12 [45]. The authors interpreted this finding as evidence that bacteria play a global role in supplying vitamins, particularly vitamin B12, to most B12-auxotrophic dinoflagellates in exchange for fixed carbon [45]. Subsequently, Tang et al. (2010) [43] reported that 91%, 49%, and 17% of studied dinoflagellates are in demand of vitamins B12, B1, and B7, respectively.

Vitamin B12 is essential for organisms, often due to incomplete or absent cobalamin-independent methionine synthase genes (*met*E). In a recently published paper, researchers examined *met*E genes from 14 strains of dinoflagellates [46]. These genes were found to be phylogenetically distinct from other known *met*E genes and uniformly lacked the complete N-terminal domains. This genetic variation provides the basis for the widespread occurrence of B12 auxotrophy in dinoflagellates.

## 5. Synergistic Utilization of Nitrogen (N) and Phosphorus (P) Nutrients by Dinoflagellates and Bacteria

Nitrogen acquisition is closely linked to the trophic modes of dinoflagellates, which exhibit remarkable complexity. Approximately 50% of dinoflagellates are photoautotrophic, while the other 50% are heterotrophic, with many species displaying mixotrophy [47]. For a long time, it was believed that bacteria were too small to be ingested by dinoflagellates. However, recent observations using fluorescence and transmission electron microscopy have revealed that multiple heterotrophic and mixotrophic dinoflagellates can indeed feed on heterotrophic bacteria and cyanobacteria [48]. Notably, 18 dinoflagellate species known to form harmful algal blooms (HABs) were observed to feed on the N_2_-fixing *Synechococcus* spp. [49,50,51]. In one study, a combination of *P. minimum* and *Prorocentrum* (*P*) *donghaiense* D. Lu, 2001 removed up to 98% of the *Synechococcus* population within just 1 h, highlighting the substantial impact of bacterial grazing by these two dinoflagellate species [51]. Although a theoretical model proposes that mixotrophic dinoflagellates meet their nitrogen requirements by ingesting cyanobacteria, this model remains untested in natural environments.

Symbiosis with diazotrophs provides dinoflagellates with a means to acquire nitrogen, especially in the open ocean where the nitrogen concentration is often limiting. Under N-limited conditions, both endosymbiosis and ectosymbiosis with cyanobacteria have been exclusively identified within the order Dinophysiales [52]. Cyanobacteria can be found either within or outside their dinoflagellate hosts [52,53]. Additionally, microscopic observations of *Ornithocercus* spp. revealed that they can ingest their symbionts, depending on the size, shape, and color of the prey, as well as the presence of a peduncle in the dinoflagellates [52,54]. However, it remains unclear whether these species primarily take up externally fixed nitrogen from cyanobacteria using transporters and only occasionally ingest the bacteria, or if they exclusively “farm” the symbionts for the purpose of feeding on them [55].

The possibility of symbiotic relationships between coral-associated Symbiodiniaceae dinoflagellates and nitrogen-fixing bacteria has drawn attention. N_2_-fixers have been identified in various coral structures, including the surface mucous layer, tissue layers, and the skeleton [56]. Notably, the amplification of the nitrogenase gene (*nif*H) in tissues of three different coral species revealed that 71% of the sequences originated from a bacterial group closely related to rhizobia—the N_2_-fixers symbiotic with legumes [57]. Additionally, studies by Lesser et al. (2007) [58] indicated that N_2_-fixation products were initially assimilated by the zooxanthellae (*Symbiodinium*) and subsequently translocated to the animal host, as confirmed by a δ^15^N analysis. Furthermore, the density of the *Symbiodinium* population was positively correlated with the copy number of *nif*H sequences, suggesting that the growth and division of zooxanthellae might depend on N_2_-fixation products [59]. Interestingly, various *Rhizobiales* bacteria have also been documented in association with other dinoflagellates, including the toxic species *Prorocentrum lima* (Ehrenberg) F. Stein, 1878 [60], *Alexandrium* (*A*) *lusitanicum* Balech, 1985 [61], *Alexandrium* (*A*) *minutum* Halim, 1960 [62], and *G. balechii* [63]. Collectively, these examples suggest that N_2_-fixing bacteria exchange their N_2_-fixing ability for protection and nutrients from their hosts, thereby providing a selective advantage to the hosts in N-limited environments.

In addition to vitamins and N, the interaction between dinoflagellates and bacteria also involves P. Phosphorus is an essential nutrient for all oceanic organisms [64,65]. The ocean’s P reservoir consists of dissolved inorganic P (DIP), primarily in the form of orthophosphate (Pi), and dissolved organic P (DOP). Phytoplankton preferentially take up Pi, while DOP is less favored [66,67]. Consequently, Pi is often depleted in the euphotic zone, limiting dinoflagellate growth due to P availability [66]. Bacteria play a crucial role in transforming different forms of DOP into the bioavailable P form for dinoflagellates. For instance, the harmful-algal-bloom-forming dinoflagellate species *P. donghaiense* can survive in P-limited environments by utilizing DOP. However, it cannot directly utilize glyphosate as a sole P source. Wang et al. (2017) [68] discovered that *P. donghaiense* was able to grow on glyphosate in the presence of glyphosate-degrading bacterial species. Notably, they amplified the *phn*J gene, which encodes the Alpha-D-ribose 1-methylphosphonate 5-phosphate C-P lyase, from the isolated bacterial community [68]. This gene is responsible for phosphonate degradation, indicating that the associated bacterial community can provide the bioavailable form of P to support the growth of *P. donghaiense* [68].

There is also competition for essential and limiting nutrients among micro-organisms, which is a recurring theme in various ecosystems. The growth of dinoflagellates is often constrained by the availability of macronutrients such as N and P. Simultaneously, bacterial activity can also be limited by these nutrients. In a study investigating the decomposition rate of freeze-dried whole cells and empty thecae of the dinoflagellate *Peridinium gatunense* Nygaard, 1925 by the microbial community, researchers discovered that intensive regenerative nutrient cycling or external nutrient inputs are necessary preconditions for efficient trophic energy transfer stored in blooms of thecate dinoflagellates [12]. This finding implies that high nutrient demands are associated with microbial degradation, indicating the competition for nutrients between heterotrophic degradative and phototrophic productive processes [12]. Furthermore, a research study conducted by Hattenrath-Lehmann et al. (2015) [69] revealed that the abundance of heterotrophic bacteria was lower in the presence of the dinoflagellate *Dinophysis* (*D*) *acuminata* Claparède & Lachmann, 1859, compared to parallel treatments without *D. acuminata*. This suggests that the alga potentially inhibits bacterial growth via nutrient competition. Specifically, N, an essential macronutrient, has been reported as a competitive resource between dinoflagellates and bacteria. For instance, a field study combining genomic, proteomic, and metabolomic approaches demonstrated that certain opportunistic bacteria with reduced genomes effectively compete for organic N compounds in coastal dinoflagellate blooms [38].

## 6. Algicidal Bacteria Inhibit the Growth of Dinoflagellates

Interactions between dinoflagellates and bacteria do not always yield beneficial outcomes; instead, they may lead to the demise of one or both partners. For example, certain bacteria are known to negatively impact dinoflagellates by inhibiting their growth or lysing algal cells [70]. These detrimental bacteria primarily belong to the CFB group or Gammaproteobacteria, such as *Muricauda*, *Pseudoalteromonas*, and *Vibrio* (Table 1). These bacteria are termed algicidal bacteria and algicidal interactions can be categorized into two practical modes: indirect or direct. In the indirect mode of algicidal interactions, the bacterium secretes dissolved chemicals that exhibited algicidal effects [71,72]. For example, an alga’s growth inhibition can be induced by an exudate collected, from which all bacterial cells have been removed through filtration [71]. Conversely, the direct mode requires live bacteria cells to effectively antagonize the alga through direct contact [73,74]. In such cases, the bacteria directly interact with the algal cells, leading to adverse consequences.

The lysis of dinoflagellate cells is the most observed effect in algicidal interactions [75,76]. Most algicidal bacteria secrete algicidal compounds to kill dinoflagellate cells, such as alkaloids, amino acid derivatives, peptides and proteins, enzymes, polyketides, terpenes, fatty acids and their derivatives, and other metabolites [72]. For example, mycosubtilins produced by *Bacillus* can interact with the cytoplasmic membrane of *C. polykrikoides*, leading to increased ion permeability and the eventual lysis of this dinoflagellate species [76]. Similarly, benzoic acid, produced by the algicidal bacterium *Thalassospira* sp., induces cell lysis in the HAB dinoflagellate *Karenia* (*Ka*) *mikimotoi* (Miyake & Kominami ex Oda) Gert Hansen & Moestrup, 2000, possibly by passing through the cell membrane and acidifying the algal cytoplasm [77]. In another study, the bacterial strain FDHY-03, belonging to the genus *Alteromonas*, targets the megacytic growth zone of *P. donghaiense* through the digestion of algal cell wall polysaccharides [78]. This process involves cell-wall-degrading enzymes, including beta-glucosidases, amylases, cellulases, and xylanases, which have been demonstrated in both laboratory and field samples as crucial factors causing algal cell lysis [78]. Additionally, other algicidal compounds, such as alkaloids, amino acids, fatty acids, and polyketides, have also been reported to exhibit algicidal activity against dinoflagellates [78,79]. On the other hand, the direct mode requires live bacterial cells to effectively antagonize the alga through direct contact [71,72]. Although limited publications have reported direct algicidal interactions between bacteria and dinoflagellates, some notable examples exist. For instance, a marine gliding bacterium, *Cytophaga* sp. strain J18/M01, successfully killed the HAB species *Gymnodinium nagasakiense* H.Takayama & M.Adachi, 1985 within a few days when they were being cultured together [80]. This bacterium likely achieves this through direct attack, as the culture filtrate—devoid of bacterial cells—had no significant effects on the growth of the same host species [80]. In the case of diatoms, the algicidal bacterium *Saprospira* sp. strain SS98-5 lyses cells of the diatom *Chaetoceros ceratosporum* Ostenfeld, 1910 through direct contact [81]. The bacterium employs gliding motility to swim toward the diatom, inducing cell aggregation [81]. Subsequently, it produces microtubule-like structures, leading to cell lysis, as observed in transmission electron micrographs [82]. While the mechanism of direct attachment in dinoflagellates remains unclear, studying diatom–bacteria algicidal interactions may provide valuable insights.

The physiological and biochemical responses of dinoflagellate cells were also detected. These include a decrease in chlorophyll *a*, the interruption of electron transport in photosystem II, reduced effective quantum yields, the accumulation of excessive reactive oxygen species, the inhibition of antioxidant enzyme activities, an increase in malondialdehyde content, and the formation of cysts [82].

**Table 1:** Algicidal bacteria recorded in publications from 2019 to 2024.

Bacterial Genus	Bacterial Class	Bacterial Phylum	Target Dinoflagellate	Reference
*Microbacterium* (1), *Brevibacterium* (4), *Bacillus* (3), *Halobacillus* (1), *Virgobacillus* (1), *Mangrovimonas* (1), *Sulfitobacter* (2), *Pelagibaca* (3), *Citreicella* (1), *Mameliella* (1), *Halomonas* (4), *Pseudomonas* (4), *Vibrio* (7), *Alteromonas* (3), *Pseudoalteromonas* (7)	Actinomycetes, Baccilli, Flavobacteriia, Alphaproteobacteria, Gammaproteobacteria	Actinobacteria, Firmicutes, Bacteroidetes, Proteobacteria	*Pyrodinium bahamense* L.Plate, 1906	[83]
*Muricauda* (2)	Flavobacteriia	Bacteroidota	*Ak. sanguinea*	[84]
*Pseudoalteromonas*	Gammaproteobacteria	Proteobacteria	*Alexandrium* (*A*) *tamarense* (Lebour) Balech, 1995	[85]
*Acetinobacter*	Gammaproteobacteria	Proteobacteria	*A. tamarense*	[86]
*Bacillus*	Bacilli	Bacillota	*A. minutum*	[87]
*Bacillus*	Bacilli	Bacillota	*Scrippsiella trochoidea* (F.Stein) A.R.Loeblich III, 1976, *Prorocentrum micans* Ehrenberg, 1834, *Peridinium umbonatum* Karsten, 1907	[88]
*Sulfitobacter*	Alphaproteobacteria	Proteobacteria	*P. donghaiense*	[74]
*Vibrio* (2)	Gammaproteobacteria	Proteobacteria	*Ak. sanguinea*	[89]
*Bacillus*	Bacilli	Bacillota	*Gy. catenatum* H.W. Graham	[90]
*Bacillus*	Bacilli	Bacillota	*C. polykrikoides*	[76]
*Cochlodiniinecator*	Alphaproteobacteria	Pseudomonadota	*C. polykrikoides*	[91]
*Paracoccus*	Alphaproteobacteria	Proteobacteria	*Ka. mikimotoi*	[92]
*Pseudoruegeria*	Alphaproteobacteria	Proteobacteria	*A. catenella*	[93]
*Stenotrophomonas*	Gammaproteobacteria	Proteobacteria	*A. tamarense*	[94]
*Sulfitobacter*	Alphaproteobacteria	Proteobacteria	*A. tamarense*	[95]
*Vibrio*	Gammaproteobacteria	Proteobacteria	*Ak. sanguinea*	[96]
*Vibrio*	Gammaproteobacteria	Proteobacteria	*Prorocentrum*	[97]
*Alteromonas*	Gammaproteobacteria	Pseudomonadota	*P. donghaiense*	[98]
*Marinobacter*, *Pseudomonas*	Alphaproteobacteria, Gammaproteobacteria	Pseudomonadota	*Ka. mikimotoi*	[99]
*Pseudoalteromonas*	Gammaproteobacteria	Pseudomonadota	*Ka. mikimotoi*	[100]
*Pseudoalteromonas*	Gammaproteobacteria	Pseudomonadota	*Noctiluca scintillans* (Macartney) Kofoid & Swezy, 1921	[101]
*Pseudoruegeria*	Alphaproteobacteria	Proteobacteria	*A. catenella*	[102]
*Shewanella*	Gammaproteobacteria	Proteobacteria	*Alexandrium pacificum* R.W.Litaker, 2014	[81]
*Shewanella*	Gammaproteobacteria	Proteobacteria	*Karlodinium veneficum* (D.Ballantine) J.Larsen, 2000	[70]
*Vibrio*	Gammaproteobacteria	Proteobacteria	*Ak. sanguinea*	[103]
*Alteromonas*	Gammaproteobacteria	Pseudomonadota	*Symbiodinium*	[104]
*Marinobacter*	Alphaproteobacteria	Pseudomonadota	*Ka. mikimotoi*	[105]
*Microbulbifer*	Gammaproteobacteria	Pseudomonadota	*Amphidinium carterae* D-044, *P. minimum* D-127	[106]
*Pseudoalteromonas*	Gammaproteobacteria	Pseudomonadota	*Ka. mikimotoi*, *A. tamarense*	[107]
*Pseudomonas*	Gammaproteobacteria	Pseudomonadota	*Gy. catenatum*, *Ka. mikimotoi*	[108]
*Qipengyuania*	Alphaproteobacteria	Pseudomonadota	*Margalefidinium polykrikoides* (Margalef) F.Gómez, Richlen & D.M.Anderson, 2017	[109]
*Shewanella*	Gammaproteobacteria	Proteobacteria	*Prorocentrum triestinum* J.Schiller, 1918	[110]
*Tenacibaculum*	Flavobacteriia	Bacteroidota	*Ka. mikimotoi*	[111]
*Arenibacter*	Flavobacteriia	Bacteroidota	*Ak. sanguinea*	[112]
*Maribacter*	Flavobacteriia	Bacteroidota	*Ka. mikimotoi*	[113]

Note: The number in brackets means the frequency of occurrence of the bacterial genus in the research paper.

## 7. The Defense of Dinoflagellate with Bacteria Challenging

Some dinoflagellates have evolved defense mechanisms to protect against unwanted bacteria. One such mechanism involves the secretion of antibacterial compounds. Notably, *P. minimum*, *Amphidinium* sp., and *Heterocapsa* (*H*) *circularisquama* Horiguchi, 1995 are reported to produce these compounds (Table 2). Among these, *Amphidinium* sp. stand out due to the diverse chemical structures of the compounds they produce, which primarily belong to the class of polyketides [114].

For instance, in a study by Kubota et al. (2014), two polyketide compounds, Amphidinin C and Amphidinin E, along with the macrolide Amphidinolide Q, were identified in the liquid medium of the cultured *Amphidinium* sp. strain 2012-7-4A [115]. These polyketide compounds exhibited antibacterial activity against *Staphylococcus* (*S*) *aureus* Rosenbach, 1884 and *Bacillus* (*B*) *subtilis* Cohn, 1872, while Amphidinolide Q specifically targeted *Escherichia* (*E*) *coli* Castellani and Chalmers, 1919 [113]. In another investigation, researchers evaluated the antibacterial activity of *A. carterae* strain LACW11 against two Gram-positive bacteria, *S. aureus* and *Enterococcus* (*En*) *faecalis* Schleifer and Kilpper-Balz, 1984 [116]. They found that Amphidinol AM-A and a new derivative, dehydroAM-A, were primarily responsible for the antibacterial effect against *S. aureus* [116].

Additionally, other antibacterial compounds from dinoflagellates include β-diketones and porphyrins, which exhibit antibacterial activity against various bacterial taxa (Table 2). Beyond polyketides, fatty acids extracted from microalgae can also inhibit bacterial growth. For example, the dinoflagellate *Lingulodinium polyedra* (F.Stein) J.D.Dodge, 1989 produces a set of fatty acids that can penetrate the cell walls of *S. aureus* and *Vibrio vulnificus* Reichelt et al., 1979, further inhibiting bacterial growth [117].

**Table 2:** A list of chemical compounds that have antibacterial activity produced by dinoflagellates.

Antibacteria Compound	Producer	Type	Bacterial Targets	Reference
1-(2,6,6-trimethy-4-hydroxycyclohexenyl)-1,3-butanedione	*P. minimum*	β-diketone	*Vibrio* sp., *Flavobacter* sp., *Chromobacterium* sp.	[118]
Luteophanol D	*Amphidinium* sp. strain Y-52	Polyketide	*Micrococcus luteus* Cohn 1872	[119]
Amphidinolide Q	*Amphidinium* sp. 2012-7-4A strain	Macrolide	*S. aureus*, *B. subtilis*, *E. coli*	[115]
Amphidinin A	*Amphidinium* sp.	Polyketide	*B. subtilis*	[120]
Amphidinin E	*Amphidinium* sp. (2012-7-4A strain)	Polyketide	*S. aureus*, *B. subtilis*	[115]
Amphidinin C	*Amphidinium* sp. (2012-7-4A strain)	Polyketide	*S. aureus*	[115]
F5	*H. circularisquama*	Porphyrin	*S. aureus*	[121]
Amphidinol dehydroAM-A	*A. carterae* strain LACW11	Polyketide	*S. aureus*, *En. faecalis*	[116]
Amphidinol AM-A	*A. carterae* strain LACW11	Polyketide	*S. aureus*, *En. faecalis*	[116]

## 8. Bacteria Involved in Dinoflagellate Toxin Production

Dinoflagellates serve as a major source of marine toxins, and bacteria are potentially involved in some of these processes, either directly or indirectly. The primary diseases caused by dinoflagellate toxins include paralytic shellfish poisoning [122], ciguatera fish poisoning [122], neurotoxic shellfish poisoning [123], azapiracid shellfish poisoning [122], and diarrhetic shellfish poisoning [122]. Additionally, other syndromes resulting from dinoflagellate-derived toxins have been reported, such as palytoxin [124] and yessotoxin [125,126].

Paralytic shellfish poisoning toxins (PSTs), produced by the *A. tamarense* species complex, consist of small heterocyclic guanidinium alkaloids. Among these, saxitoxin (STX) serves as the basic form, with 58 other analogs documented [127]. A hypothesis has been formulated suggesting that the co-cultured bacteria within or associated with the toxic *A. tamarense* species complex are responsible for STX production [128]. Numerous studies have investigated this hypothesis and have provided a range of findings—from the production of PSP by isolated intracellular and free-living bacteria to no direct involvement of bacteria in PSP toxin production. For instance, in Doucette and Trick (1995) [129], a putatively toxic bacterium within the toxic dinoflagellate *A. tamarense* was investigated for its ability to synthesize PSTs. High-performance liquid chromatography analyses revealed that this bacterium produced PSTs regardless of the nutritional status of the host dinoflagellate [129]. Additionally, a bacterium isolated from the cultured medium of *Protogonyaulax tamarensis* (Lebour) F. J. R. Taylor, 1979 contained STX capable of killing mice [128]. However, other studies have yielded conflicting results regarding the role of bacteria in the origin of STX. For example, Green et al. (2004) [130] demonstrated that nine bacterial strains isolated from the PST-producing *Gy. catenatum* culture did not produce compounds with PST-like activity. Similarly, in Martins et al. (2003) [131], two bacterial strains isolated from toxic dinoflagellates were evaluated using biological and analytical methods, and no PST production was detected. Recent RNA-sequencing results also revealed the existence of some *sxt* genes with poly-A tails and spliced leader sequences in *A. minutum* and *Alexandrium fundyense* Balech, 1985 [132]. These findings may indicate that bacteria do not play a direct role in PST production by the *A. tamarense* species complex. However, it is also possible that specific bacterial types were inadvertently removed during various procedures. Additionally, previous studies using methods to measure bacterial metabolites may have lacked specificity, leading to conflicting results. Some compounds initially thought to be STX were later identified as chemical imposters [127,131,133].

Beyond their direct involvement in dinoflagellate toxin synthesis, some bacteria may either enhance or inhibit the toxicity of their associated partners. For instance, in a study by Uribe and Espejo (2003) [134], axenic cultures of *A. catenella* were still able to produce toxins after the removal of saprophytic bacteria but the total toxicity diminished to about one-fifth of that observed in non-axenic cultures. In another study by Wu et al. (2022) [21], bacterial strains of *Oceanococcus* and *Marinoscillum* exhibited a strong positive correlation with the toxin production of *G. balechii*. Furthermore, Wang et al. (2018) [135] investigated the regulatory roles of quorum-sensing (QS) bacteria in the toxicity of the CFP-causing *Gambierdiscus* spp. Their results indicated that the algal host generally displayed much a higher toxin production ability in the presence of QS strains. Notably, *Bacillus anthracis* had a remarkable influence on *Gambierdiscus* toxicity [135]. It is plausible that the increased toxicity serves as a self-protective strategy, empowering the algae with a greater competitive ability in a deteriorative environment. Consequently, the presence of multiple species-specific bacteria could induce algal responses, such as toxin production, for self-protection.

## 9. Other Interactions

Sometimes, the association between dinoflagellates and bacteria is more complex than we documented above. For instance, their relationship can switch between mutualistic and pathogenic lifestyles. In a study conducted by Wang et al. (2015) [136], they observed a typical “Jekyll and Hyde” scenario. Specifically, they co-cultured the toxic dinoflagellate *P. minimum* and the Alphaproteobacterium *Dinoroseobacter shibae* Biebl et al., 2005 in a mineral medium lacking a carbon source and vitamins for the bacterium, as well as the essential vitamin B12 for the dinoflagellate [136]. Initially, both *P. minimum* and *D. shibae* exhibited an increase in growth but then the mutualistic phase was followed by a pathogenic phase during which the bacteria induced the death of the algae [136]. In summary, the relationship between dinoflagellates and bacteria is dynamic, with transitions between co-operation and antagonism depending on environmental conditions and nutrient availability.

## 10. Bacteria in Dinoflagellate Genome Evolution

Dinoflagellates represent an evolutionarily unique group with novel genomic characteristics. One distinctive feature is their possession of large nuclear genomes, ranging from approximately 1.75 to 268 gigabase pairs of DNA [137,138,139]. These genomes are organized into permanently condensed liquid-crystalline chromosomes, numbering from several to over 100 [47]. Remarkably, dinoflagellates are gene acquirers, drawing from a diverse pool of organisms, including peridinin dinoflagellates, streptophytes, heterokonts, red algae, green algae, diatoms, viruses, bacteria, and other unknown sources [47,140,141,142]. These genes were acquired through either endosymbiotic gene transfer (EGT) or horizontal gene transfer (HGT). While HGT is well-established as a major driver of genetic innovation in bacteria and archaea, its relative importance in the evolution of eukaryotes remains unsettled [143]. Recent advances in sequencing technology, from Sanger sequencing to PacBio SMRT sequencing, have enabled phylogenomic analyses that reveal an increasing number of genes transferred from bacteria into the genomes of microbial eukaryotes. Examples include green algae, apicomplexans, ciliates, and fungi [144,145,146,147,148]. This process also likely contributes to some of the most distinctive features of dinoflagellate biology. However, quantifying the specific number of bacterial genes in dinoflagellate genomes remains challenging, given their enormous genome sizes.

A total of 34 genes of possible bacterial source were documented in this review and these genes might be acquired from cyanobacteria, Proteobacteria, and Bacteroidetes (Table 3). The first HGT case in dinoflagellates from bacteria was reported by Whitney et al. (1995) [149] in which the authors found that the peridinin-containing dinoflagellates use the form II ribulose-1,5-bisphosphate carboxylase-oxygenase (RuBisCO) of proteobacterial origin. RuBisCO is an enzyme involved in photosynthesis and the most usual form of RuBisCO in chloroplasts is form I [150]. Unlike other photosynthetic eukaryotes, the form II RuBisCO in dinoflagellates is not encoded in the chloroplast genome but rather by the nuclear DNA [151]. Rhodopsin is another case of HGT reported in dinoflagellates (Table 3). Proteorhodopsins (pRhodopsin) are photoactive membrane proteins that utilize retinal as a chromophore for light-mediated functionality [152]. Initially discovered in bacteria, pRhodopsins have also been reported in archaea and eukaryotic micro-organisms, such as dinoflagellates, which acquired multiple types of rhodopsin through HGT from bacteria [153,154]. In a study by Slamovits et al. (2011) [155], 96 rhodopsin protein sequences representing the known diversity of microbial rhodopsins across three domains of life were used to generate a maximum-likelihood phylogenetic tree. Three major dinoflagellate groups were identified: one related to sensory-type rhodopsins from cryptomonad algae, halophilic archaea, and fungi; and the other two groups related to marine bacteria [155]. This finding suggests that dinoflagellates in these two groups acquired rhodopsin genes via HGT from bacteria. Furthermore, bacteria are possibly involved in toxin production in dinoflagellates (described above), and they may also have genetic contributions. For example, *sxt*A [132] and *sxt*G [127] of bacterial origin in the dinoflagellate genome are likely related to the STX toxin production, whereas PKS/NRPS of Burkhoderiales origin might be responsible for the polyketide synthesis in dinoflagellates [156]. Other HGT cases include the *aro*B and O-methyltransferase genes (Table 3) which are from cyanobacteria and move into dinoflagellates between the divergence of *Perkinsus* and *Oxyrrhis* [142]. These two genes fused and formed a novel plastid-targeted gene, which is not found in any other eukaryotic lineage [142].

Dinoflagellates employ several possible mechanisms for acquiring foreign genes. One such mechanism is the obligate endosymbiosis, a stable form of physical association. Examples of obligate endosymbiosis include the evolution of mitochondria and plastids from bacterial endosymbionts [161,162,163]. Additionally, many other endosymbionts have also contributed genetic material to their host genomes. A remarkable case of HGT was identified in the peridinin plastid genome of dinoflagellates. This peridinin plastid genome is organized into plasmid-like minicircles [164]. Genomic and phylogenetic analyses of minicircles from *Ce. horridum* and *Py. lunula* revealed four genes and one unannotated open reading frame, likely acquired from bacteria belonging to Bacteroidetes [164]. The close endosymbiotic associations observed between these bacteria and dinoflagellates suggest that this is indeed an HGT case arising from endosymbiosis. Phagotrophy, the process of feeding on other organisms, is widespread in dinoflagellates. This aligns with the concept proposed by Doolittle in 1998: the “you are what you eat” gene transfer ratchet [165]. Evidence of HGT has been documented in phagotrophic lineages such as ciliates [148], euglenids [166,167], and amoebae [168] Given the prevalence of phagotrophy in dinoflagellates, it is plausible that feeding on bacteria serves as another avenue for these organisms to acquire genetic material of bacterial origin.

The acquisition of new genes or functionally related gene sets can significantly benefit dinoflagellates. Firstly, HGT acts as a crucial source of gene diversity in these organisms. Secondly, HGT plays a pivotal role in their adaptation to environmental conditions (such as *sxt*A and *sxt*G genes). Additionally, HGT has been implicated in the exploitation of new ecological niches by dinoflagellates (such as the form II RuBisCO gene). Bacteria likely continue to contribute numerous genes to the dinoflagellate genomes, and further research will shed light on these fascinating evolutionary processes.

## 11. Biofilms

A biofilm is a community of micro-organisms that can attach to surfaces of plants [169], phytoplankton [170], and other abiotic surfaces [171]. These microbial cells become embedded within a matrix of highly hydrated extracellular polymeric substances (EPSs) [172]. The EPSs consist of proteins, polysaccharides, and extracellular DNA (eDNA) [173]. Biofilms formed by bacteria associated with higher plants can either promote plant health or trigger pathogenesis and have been thoroughly investigated [174]. Similarly, in aquatic ecosystems, bacterial biofilms can benefit their microalgal hosts by providing nutrients or harm them by secreting algicidal compounds [175,176]. However, there are limited reports on the interaction between dinoflagellates and biofilm-forming bacteria. One study showed that free-living *Symbiodinium* spp. in culture commonly form calcifying bacterial–algal biofilms that produce aragonitic spherulites and encase the dinoflagellates as endolithic cells [177]. This process is driven by *Symbiodinium* photosynthesis but occurs only in partnership with bacteria, highlighting the important role of dinoflagellates and associated microbial biofilms in the organomineralization process in marine ecosystems [177]. Given the common ecological principles that govern the assembly of microbial communities in the algal phycosphere and the rhizosphere of higher plants [178], we believe that in-depth research on the interaction between dinoflagellates and bacteria from the perspective of biofilm function is a crucial research direction.

## 12. Conclusions

The common features of the interaction between dinoflagellates and bacteria were summarized in this review. It particularly emphasizes the bacterial taxa that are consistently identified in association with dinoflagellate monocultures or environmental samples, notably, Alphaproteobacteria, Gammaproteobacteria, and members of the Cytophaga–Flavobacterium–Bacteroides complex. The interactions between dinoflagellates and bacteria are characterized by the exchange of substances such as dissolved organic matter, vitamins, nitrogen, and phosphorus, as well as the mutual secretion of pathogenic agents, including algicides and antibacterial substances. Additionally, bacteria play a pivotal role in the biosynthesis of dinoflagellate-derived compounds, such as toxins. Moreover, bacteria are instrumental in the evolutionary trajectory of dinoflagellates by transferring numerous genes into their genomic repertoire. Finally, the biofilms developed around the dinoflagellate cells by bacteria may play important roles in the organomineralization process in ocean.

## 13. Future Directions

In this review, we discussed the complex interactions between dinoflagellates and bacteria within the marine ecosystems. We summarized the current knowledge regarding the physiological, ecological, and evolutionary dynamics of their association. Nevertheless, there are more questions than answers, especially when the environment of their coexistence is dynamic. Key inquiries include: (1) the functional role of specific bacteria within the core microbiome during interaction; (2) the mechanisms governing the transfer of substances they exchange; (3) the presence of signaling pathways that trigger the production of pathogenic agents; (4) the impact of horizontally transferred genes from bacteria on the evolutionary development of dinoflagellate characteristics; and (5) the interaction between dinoflagellates and bacteria from the perspective of biofilm function. To address these complexities, a more detailed investigation and more advanced approaches are needed.

## Figures and Tables

**Table 3:** HGTs found in dinoflagellates.

Gene	Enzyme	Dinoflagellate Source	Localization	Bacterial Origin	Bacteria Phylum	Reference
*ace*E	Pyruvate dehydrogenase	Dinoflagellates	Genome	-	Actinobacteria	[15]
*aro*B	3-dehydroquinate synthase	*Oxyrrhis*	Genome	-	Cyanobacteria	[142]
OMT	O-methyltransferase	*Oxyrrhis*	Genome	-	Cyanobacteria	[142]
*asl*A	Arylsulfatase A	*Karenia* (*Ka*) *brevis* (C.C.Davis) Gert Hansen & Moestrup, 2000	Genome	-	-	[157]
ATS1	Alpha-tubulin suppressor	*Ka. brevis*	Genome	-	-	[158]
*avt*A	Valine:pyruvate aminotransferase	Dinoflagellates	Genome	-	Actinobacteria	[15]
CAS-like	Clavaminic acid synthetase-like protein	*Ka. brevis*	Genome	-	Cyanobacteria	[157]
*cit*E	Citrate lyase beta subunit	Dinoflagellates	Genome	-	Proteobacteria	[15]
Epimerase	NAD dependent epimerase/dehydratase	*Ka. brevis*	Genome	-	-	[157]
Fe-ADH	Iron-containing alcohol dehydrogenase	*Ka. brevis*	Genome	-	-	[157]
Form II Rubisco	Form II ribulose-1,5-bisphosphate carboxylase-oxygenase	Peridinin-containing dinoflagellates	Genome	-	Proteobacteria	[149]
*grp*E	Protein GrpE	Dinoflagellates	Genome	-	-	[15]
HLP	Histone-like protein	*A. tamarense*	Genome	-	-	[149]
*lig*I	Metal-dependent hydrolase, TIM-barrel fold	*Ka. brevis*	Genome	-	-	[157]
MQO	Monomeric NADP(+)-dependent isocitrate dehydrogenase	*Ka. brevis*	Genome	-	-	[158]
MVIM	MVIM-sugar aminotransferase	*Ka. brevis*	Genome	-	Proteobacteria	[157]
*pbp*B	Substrate-bound, membrane-associated, periplasmic binding protein	*Ka. brevis*	Genome	-	-	[157]
*pdx*A	Pyridoxal phosphate biosynthetic protein	*Ka. brevis*	Genome	-	-	[157]
*ptdss*	Phosphatidylserine synthase	Dinoflagellates	Genome	-	Proteobacteria	[15]
*put*A	NAD-dependent aldehyde dehydrogenases	*Ka. brevis*	Genome	-	-	[157]
RHO	Rhodopsin synthesis	*Oxyrrhis* (*O*) *marina* Dujardin, 1841	Genome	-	-	[155]
*rlm*F	SAM-dependent methyltransferase	*Ka. brevis*	Genome	-	-	[157]
*rpl*28	60S ribosomal protein L28	*Pyrocystis* (*Py*) *lunula* (Schütt) Schütt, 1896	Plastid genome	*Cytophaga*	Bacteroidetes	[158]
*rpl*33	Large ribosomal subunit protein bL33c	*Py. lunula*	Plastid genome	*Cytophaga*	Bacteroidetes	[158]
SIR2	Silent information regulator 2	*Ka. brevis*	Genome	-	Proteobacteria	[157]
SRP54 N domain	The signal recognition particle 54-kDa subunit	*Pyrocystis*	Plastid genome	-	Bacteroidetes	[158]
*sxt*A	8-amino-7-oxononanoate synthase	*Alexandrium* and *Pyrodinium*	Genome	-	-	[128]
*sxt*G	Glycine amidinotransferase	*Alexandrium* species and *Gy. catenatum*	Genome		-	[127]
WECE	Pyridoxal phosphate dependent aminotransferase	*Ka. brevis*	Genome	-	Proteobacteria	[157]
*yaa*A	DNA-binding and peroxide stress resistance	*Ka. brevis*	Genome	-	-	[157]
*ycf*16	Probable ATP-dependent transporter ycf16	*Ceratium* (*Ce*) *horridum* (Cleve) Gran, 1902	Plastid genome	*Algoriphagus*	Bacteroidetes	[157]
*ycf*24	Iron-sulfur cluster assembly SufBD family protein ycf24	*Ce. horridum*	Plastid genome	*Algoriphagus*	Bacteroidetes	[158]
MCA	Metacaspase	*C. polykrikoides*	Genome	-	-	[159]
PKS/NRPS	Non-ribosomal peptide synthases/polyketide synthases	*O. marina* and core dinoflagellates	Genome	*Burkholderiales*	Pseudomonadota	[156]

Note: Dash means that the specific species has not been elucidated. Larger-scale analysis also provides insights on the HGT cases from bacteria. For example, ESTs revealed a total of 50 genes that were possible cases of HGT in *Symbiodinium* as they had previously only been identified in bacteria or other dinoflagellates [160]. These genes encoded proteins that were involved in a variety of functions including carbohydrate transport, inorganic ion transport, stress response, and proteases, amongst others [160]. In Wisecaver et al. (2013) [15], the comparison of gene set between *A. tamarense* Group IV and 16 other eukaryotic genomes showed that *A. tamarense* Group IV has the largest number of gene families gained. Phylogenomic analysis indicates that genes horizontally acquired from bacteria are a significant portion for those dinoflagellates grouped with bacteria in gene phylogenies for 92 (10.5%) KEGG-annotated *A. tamarense* Group IV contigs.

## Data Availability

Not applicable.
